# Pulmonary Symptoms After Mild COVID-19: A Retrospective Observational Study

**DOI:** 10.7759/cureus.84499

**Published:** 2025-05-20

**Authors:** Megan Koster, Lisa D Moore, Lanny S Inabnit

**Affiliations:** 1 Respiratory Care, Boise State University, Boise, USA; 2 Pulmonary Function, Arizona Pulmonary Specialists, Ltd., Scottsdale, USA

**Keywords:** diagnostics, long covid, long covid-19 pulmonary sequelae, persistent covid-19 symptoms, post-covid complications, pulmonary function test

## Abstract

Background

Patients have reported persisting or emerging symptoms weeks or months after the acute phase of a COVID-19 infection, even when the initial infection was mild or asymptomatic. Since data are limited on patients with a history of a mild acute phase, the pulmonary sequelae have been challenging to treat. The purpose of this study was to review diagnostic testing sessions from patients referred to the pulmonary function lab for a diagnosis or symptoms of long COVID following a mild acute phase. The collection and presentation of this data aim to provide insight into the possible cause of persistent pulmonary symptoms in this specific patient population.

Methodology

A retrospective review of patients at a single center who received pulmonary function test (PFT) and 6-minute walk test (6MWT) for long COVID symptoms following a mild acute phase of COVID-19 was conducted. Adult subjects were identified through electronic medical records if they received services between March 1, 2020, and September 2, 2023. The records of 19 patients were included in this review.

Data analysis

A Pearson correlation coefficient was used to analyze the relationship between dyspnea, pulmonary function impairment severity, and fatigue. The relationship between dyspnea and 6-minute walk test distance (6MWTD) was analyzed using the phi correlation coefficient.

Results

Weak correlations were identified between the reported symptoms of dyspnea and the severity of airway impairment, gas exchange, and 6MWTD. There was a strong positive correlation between dyspnea and fatigue. The sample size limits the analysis, but the data substantiate the need for additional research.

Conclusion

The severity of lung function impairments did not appear to correlate with the severity of dyspnea in patients following a mild acute phase of COVID-19. Therefore, pulmonary function impairments did not appear to be the primary cause of dyspnea. However, the symptoms of dyspnea and fatigue may contribute to each other.

## Introduction

Since the beginning of the severe acute respiratory syndrome coronavirus 2 (SARS-CoV-2) pandemic, patients experiencing lingering symptoms have been documented [1]. The National Institutes of Health (NIH) refers to the condition as post-acute sequelae of SARS-CoV-2 infection or long COVID [2]. The definition includes ongoing, relapsing, or new symptoms four or more weeks after the acute infection [2]. The Department of Health and Human Services (DHHS) asserted that 51% of those surveyed had to reduce their working capacity due to symptoms of long COVID and 44% could not return to work. The condition is roughly estimated to cause $50 billion in annual salary loss [2]. To mitigate the impact, DDHS created the National Research Action Plan on Long COVID. This document calls for researchers to gather data on long COVID to accelerate understanding.

The primary self-reported pulmonary sequela is dyspnea on exertion [1,3]. Bouteleux et al. and Gloeckl et al. attempted to demonstrate that the condition could be treated with pulmonary rehabilitation [1,4]. However, despite some measurable improvements, patients continued to experience dyspnea. Early evidence suggested that sequelae symptoms persisting for 12 months were more common in patients who had been critically ill. While the severity level during the acute phase appeared to increase the risk of prolonged impairments, patients who were initially asymptomatic or experienced a mild acute phase also reported long COVID symptoms [2,3,5,6]. Unfortunately, data on this patient population with a history of a mild acute phase of COVID-19 are limited. Therefore, there is a need to better understand the mechanisms of dyspnea in this population.

The purpose of this study was to review pulmonary function tests (PFTs) and 6-minute walk tests (6MWTs) of individuals who received testing due to long COVID symptoms following a mild acute phase. The investigation focused on measurable pulmonary function parameters, 6-minute walk test distance (6MWTD), and subjective symptoms of dyspnea and fatigue. The study aimed to identify whether there was a correlation between dyspnea, the primary pulmonary sequela, and abnormal pulmonary function measurements [1,3]. Secondly, the correlation between the self-reported subjective symptoms of dyspnea and fatigue was also evaluated. The objective was to gain insight into the potential mechanisms of dyspnea in this understudied population, which may inform further research.

This article was previously published as an abstract in an online supplement to the October 2024 issue of Respiratory Care. Additionally, it was presented as a poster at the 2024 American Association for Respiratory Care Open Forum on November 22, 2024, in Orlando, Florida. The Boise State University Independent Review Board approved the ethics of the study.

## Materials and methods

The electronic health record (EHR) was searched to obtain a list of patients who underwent PFT and 6MWT to evaluate persistent symptoms following a mild acute phase of COVID-19. The ICD-10-CM diagnosis codes used to search the EHR were post-COVID-19 condition unspecified (U09.9) and personal history of COVID (Z86.16). Records for patients matching these inclusion criteria and who were seen between March 1, 2020, and September 2, 2023, were included in this investigation.

To meet the definition of long COVID, symptoms had to have persisted or progressed for over four weeks after the initial infection [2]. A mild acute phase was defined as requiring little to no intervention or no hospitalization. These patients' self-reported acute phase symptoms resembled an upper respiratory tract infection or included no notable events. Descriptions of the acute phase included such statements as "felt poorly," "did not need any medications," "mild cough," "irritating cough," and "did not need hospitalization."

Study population

**Figure 1:**
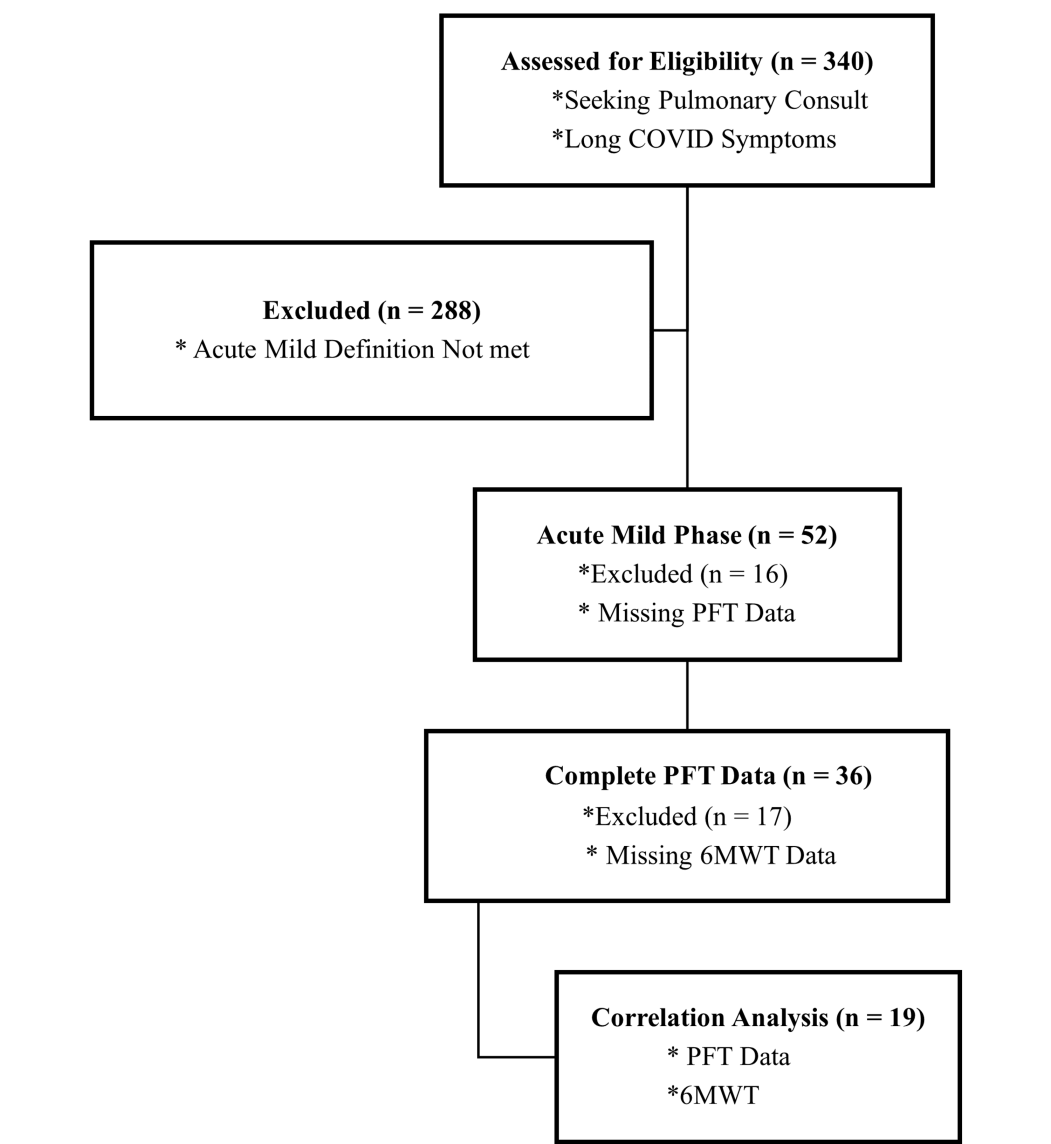
Attrition diagram PFT, pulmonary function test; 6MWT, 6-minute walk test

The EHR search identified 340 adult patients seeking a pulmonary consultation for persistent symptoms post-COVID-19 or long COVID between March 2020 and September 2023. Among this sample, 52 patients met the criteria for having a mild acute phase. Following the initial consultation with the pulmonologist, complete PFTs were ordered for 36 patients. Of these patients, 19 also had a 6MWT ordered by the pulmonologist. The subjects who did not have a complete PFT and a 6MWT were eliminated from the correlation analysis due to missing information; Figure 1 illustrates the patient selection process.

Many of these patients had preexisting conditions but most had never experienced breathing issues that prompted them to seek care from a pulmonologist before. Years ago, one patient sought the care of a pulmonologist for an unrelated breathing issue but did not receive continued care. Before their COVID-19 infection, none of the included patients had breathing issues that prompted them to pursue pulmonary diagnostic testing. However, following an acute phase of COVID, they experienced persistent pulmonary sequela that led them to seek an evaluation from a pulmonary specialist. Consequently, the patients included in the analysis met the Department of Health and Human Services (2022) definition of long COVID, met the inclusion criteria of a mild acute infection, were new patients to the clinic, and had no previous pulmonary diagnostic testing.

Data collection

The patient's perception of dyspnea and fatigue was measured using the modified Borg scale after exertion from the 6MWT [7]. The distance achieved during the 6MWT was measured in meters and then compared to a predicted lower limit of normal (LLN) for a healthy adult [8]. The equations developed by Enright and Sherrill were used to determine the predicted distance and establish the LLN. Accordingly, 6MWTD was categorized as normal if the patient met a 6MWTD greater than or equal to their predicted LLN. The results were considered abnormal if the 6MWTD was less than the predicted LLN.

PFT measurements were obtained with the Platinum Elite Series™ Plethysmography equipment (MGC Diagnostics, Saint Paul, MN). PFT included spirometry, static lung volumes via plethysmography, and lung diffusing capacity of carbon monoxide (DLCO). Dynamic and static lung volumes were interpreted in the context of each other, providing a more complete assessment of overall pulmonary function [9,10]. These measurements were used to identify airway impairments indicated by patterns of obstruction or restriction. The DLCO measurements were used to identify gas exchange impairments. PFT results were categorized according to impairment severity levels [11].

Statistical analysis

The Borg scale's numeric scores given by patients were used to determine the intensity level of dyspnea and fatigue. The interpreting physician determined the degree or level of severity assigned to pulmonary function impairments. The interpretation strategies used in the clinic most commonly align with the recommendations from Pellegrino et al.'s 2005 publication [11]. The severity levels of pulmonary function (PF) impairments were assigned a numeric value. The numerical assignments were as follows: normal = 0; mild = 1; moderate = 2; severe = 3; very severe = 4. The numerical assignments allowed for a Pearson correlation analysis to determine the strength of relationships between dyspnea scores and PF interpretations [12]. A Pearson correlation was used to measure the degree of linear correlation between dyspnea and fatigue Borg scores [12]. The symptoms of dyspnea were also categorized as either “yes” or “no” to perform a phi correlation to measure the strength of the association between dyspnea and an abnormal 6MWTD. The 6MWTD was categorized as either normal or abnormal.

## Results

The age range for these patients was 30 to 81 years, with an average (mean) patient age of 60.11 years (standard deviation 13.45). The patient demographics for this sample were as follows: 53% of the patients were male, 53% had a history of smoking, and 89% had some type of preexisting condition, including asthma, hypothyroidism, fibromyalgia, diabetes, and various cardiac issues (Table 1).

**Table 1:** Demographics and clinical characteristics of the cohort

Patient	n	%
Age (years), mean (SD)	60.11 (13.45)	
Male (birth sex), N (%)	10	53
Former smoker, N (%)	10	53
Comorbidities, N (%)	17	89

When the spirometry and lung volumes were considered in the full context of each other, the complete PFT profile of this sample (Table 2) revealed that 32% had “normal” PFT results, 16% were classified as having a mild restriction, and 47% had a mild obstruction. Mild diffusion defects were found in five (26%) of patients. A combination of mild obstruction and mild diffusion defect was present in 15% of tests, and 0.05% had a combination of mild restriction with a mild diffusion defect.

**Table 2:** PFT interpretation PFT, pulmonary function test

Severity of Impairment	n	%
Normal	6	32
Mild restriction	3	16
Mild obstruction	9	47
Mild diffusion defect	5	26
Combined obstruction/diffusion	3	16
Combined restriction/diffusion	1	<1

Approximately 80% of the participants reported some sensation of dyspnea, with the severity ranging from moderate to severe, while 21% reported no dyspnea (Table 3). Thirty-two percent of patients achieved a distance greater than or equal to their predicted LLN, which indicates a normal testing performance. The remaining 68% completed a distance less than their predicted LLN, which indicates an abnormal or reduced functional capacity [7]. Following the 6MWT, patient fatigue severity was measured using the Borg scale [7]. Twenty-six percent of patients reported feeling no fatigue at all. Eleven percent described their fatigue as very slight. However, 63% of patients reported slight to severe fatigue (Table 4).

**Table 3:** End-6MWT dyspnea Borg scale 6MWT, 6-minute walk test

Borg Score	n	%
0 (nothing at all)	4	21
0.5 (just noticeable)	1	<1
1 (very slight)	0	0
2 (slight)	3	16
3 (moderate)	5	26
4 (somewhat severe)	3	16
5 (severe)	3	16

**Table 4:** Fatigue assessment using the Borg scale

Borg Score	n	%
0 (nothing at all)	5	26
0.5 (just noticeable)	0	0
1 (very slight)	2	11
2 (slight)	5	26
3 (moderate)	4	21
4 (somewhat severe)	0	0
5 (severe)	3	16

**Figure 2:**
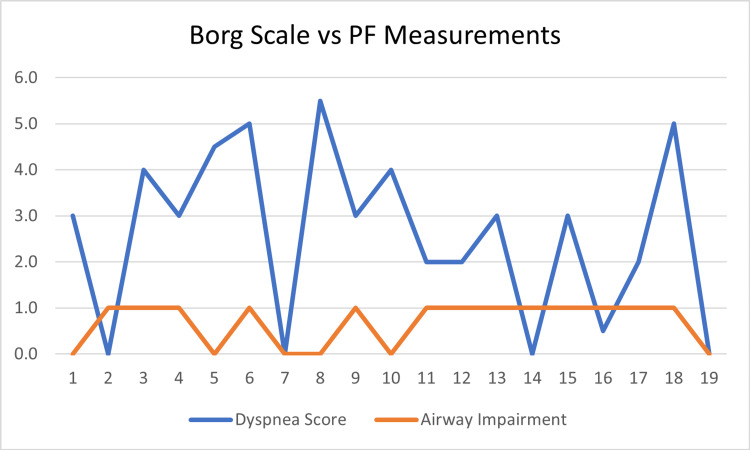
Dyspnea and airway impairments PF, pulmonary function

**Figure 3:**
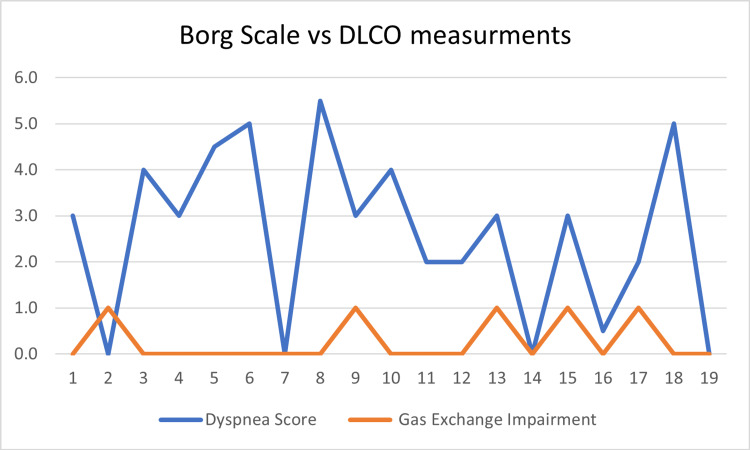
Dyspnea and gas exchange impairments DLCO, diffusing capacity of the lungs for carbon monoxide

**Figure 4:**
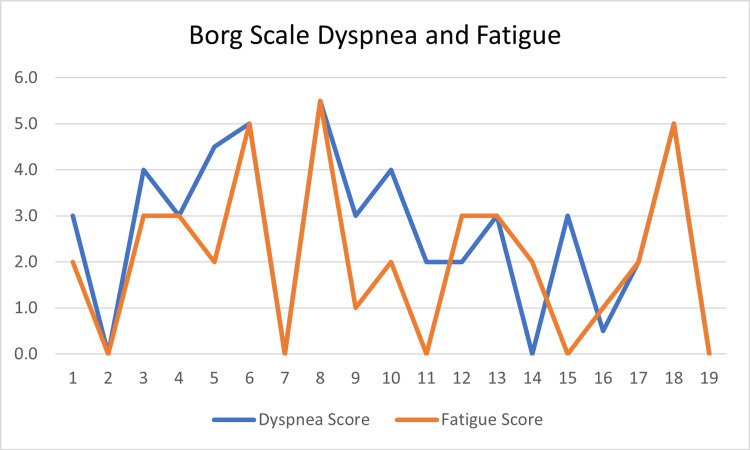
Dyspnea and fatigue

Table 5 presents the relationship between dyspnea and the following variables: airway, gas exchange, fatigue, and 6MWTD. A weak, negative relationship was found between the severity of PF airway impairment and the severity of dyspnea (r (17) = -0.09, p = 0.72) (Figure 2). This finding was not statistically significant, with the significance level set at a p-value of 0.05. There was also a weak relationship between the severity of gas exchange impairment and the reported severity of dyspnea (r (17) = -0.14, p = 0.58) (Figure 3). Additionally, no significant relationship was found between experiencing symptoms of dyspnea and achieving a lower-than-normal 6MWTD (r (1) = 0.05, p = 0.37). There was, however, a significantly strong relationship between reported symptoms of dyspnea and feelings of fatigue (r (17) = 0.75, p = 0.00) (Figure 4).

**Table 5:** Pearson and phi correlation results LLN, lower limit of normal; 6MWTD, 6-minute walk test distance

Dyspnea vs. Impairment	Coefficient (r)	Relationship	p-value
Airway	-0.09	Weak	0.72
Gas exchange	-0.14	Weak	0.58
Fatigue	0.75	Strong	<0.00
< LLN 6MWTD	0.05	Weak	0.37

## Discussion

The lack of correlation between the measurable PF parameters indicating patterns of airway or gas exchange impairment and subjective symptoms is consistent with the existing literature. According to the Global Initiative for Chronic Obstructive Lung Disease guidelines, there is only a weak correlation between patients' symptoms and the spirometric severity of airflow obstruction. Clinicians should be mindful that the severity of the disease can differ from measured impairments [13]. Although this study did not focus on patients with chronic obstructive pulmonary disease, 47% were found to have a mild obstruction. It is possible that the mild airflow impairments found did contribute to the patient's discomfort, despite a weak correlation with the intensity of their shortness of breath. Future research on the reversibility of airflow obstruction with the use of bronchodilation medications could prove to be valuable.

The Borg scale addresses the patient's subjective feelings, level of discomfort, and intensity of symptoms. However, similar to PF measurements, the Borg scale does not explicitly indicate how the symptoms impact the quality of life or alter behavior [14]. Conversely, the 6MWTD reflects the exercise tolerance required to perform activities of daily living [14]. Overall, 68% of our subjects had a 6MWTD below their LLN. This reduced functional capacity suggests a reason for concern [7].

Mancini et al. and Singh et al. performed cardiopulmonary exercise testing (CPET) on patients with normal PFT results but experiencing persistent dyspnea [15,16]. Abdallah et al. performed CPET on patients with mild PF impairments and persistent dyspnea [6]. These studies demonstrated that patients had reduced oxygen consumption (VO2) and hyperventilatory respiratory patterns, leading the authors to conclude that the most likely issue was an impairment in peripheral perfusion. The presence of extrapulmonary impairment could explain why the cause of dyspnea could not be identified during a PFT.

This research did not include CPET; however, a 6MWT will elicit a VO2 peak similar to CPET [7]. Although during a 6MWT, the VO2 peak is reached more gradually, a reduction would result in decreased performance [7]. Therefore, the research from Mancini et al., Singh et al., and Abdallah et al. could explain why 68% of this cohort had an abnormal 6MWTD, 79% reported some level of dyspnea, and 74 % reported some level of fatigue following a self-paced walk on a flat surface. Forthcoming research and treatment of this patient population should consider first obtaining CPET to determine if the patient's symptoms are more likely the result of cardio or pulmonary limitations. This could reduce the number of diagnostic tests the patient may undergo. It could decrease the frustration and costs associated with diagnostic testing that provides minimal information.

Limitations

The inclusion criteria and the single center yielded a small sample size, limiting the results' generalizability. Additionally, none of the subjects included in the investigation had any previous PFT or 6MWT testing before their acute COVID-19 infection. Because there was a lack of baseline testing, the mild impairments identified could have been present before the acute illness. The effects of confounding variables, such as pre-existing comorbidities, cannot be ruled out. Lastly, the 6MWT in this facility is performed in a 50-foot hallway instead of a 100-foot hallway. The additional turns required can decrease the distance the patients achieve [7].

## Conclusions

The study aimed to explore the relationships among persistent pulmonary sequelae following a mild course of COVID-19. Only weak correlations were identified between the subjective symptom of dyspnea and PF measurements and 6MWTD. However, a strong correlation was found between the subjective symptoms of dyspnea and fatigue. It appears that the sample of patients in this investigation was experiencing symptoms related to pulmonary sequalae, but the primary cause remains unclear. These observations indicate that the degree of breathlessness experienced by some patients who fit the DHHS definition of long COVID is most likely not occurring from PF impairments. Future research investigating symptoms following a mild case of COVID-19 should focus on alternative diagnostic testing and therapeutic options.
